# Academy of Stomatology

**Published:** 1904-04

**Authors:** 

**Affiliations:** Academy of Stomatology


### Reports of Society Meetings.

### ACADEMY OF STOMATOLOGY.

A REGULAR meeting of the Academy of Stomatology of Philadelphia was held at the rooms of the Academy, 1731 Chestnut Street, on the evening of Tuesday, November 24, 1903, the President, Dr. L. Foster Jack, in the chair.

A paper was read by Dr. R. M. Sanger, of East Orange, N. J., entitled "The Diatoric Tooth in Bridge-Work."

(For Dr. Sanger's paper, see page 233.)

DISCUSSION.

*Dr. H. Roberts.*—I have been very much interested in the doctor's method, which seems to cover ground very improperly covered heretofore. One of the greatest objections I have had to bridge-work is that the occlusal surface had to be of gold; otherwise, one is liable to have a broken surface very difficult to repair. I am heartily in sympathy with the essayist in regard to devitalizing an anterior tooth instead of putting a jacket on it, which I think is almost as bad as a full gold crown,—as the cement will wash out underneath. The method of covering the palatine surface in conjunction with a pin in the root furnishes a good support for an anterior bridge. Adjusting the caps by means of wax is very simple, and you can get a better adjustment in that way than by a cast. There are some few cases where I think a gold crown on a front tooth the only thing to be used with safety, although, as a rule, I condemn its use in this position.

*Dr. P. B. McCullough.*—I would like to ask Dr. Sanger how, in uniting the tooth with the gold, he prevents the sulphur taking fire.

*Dr. Sanger.*—The sulphur is on the inside of the cap and the flame on the other side, so that the flame does not touch the sulphur. Of course, one must be careful not to overheat it.

*Dr. C. R. Jefferis.*—In replacing the porcelain, would the doctor use oxyphosphate?

*Dr. Sanger.*—I would repair with oxyphosphate. There is no difficulty in holding these teeth with it, as the amount of exposed surface is very small. The action of the saliva on the oxyphosphate would be slight.

*Dr. A. W. Jarmon.*—I am in accord with the system and the cornmeal method of swaging. The ordinary facing with the pins bent can be held in position on an ordinary bridge with oxyphosphate. This is better than simply to have the pin bent.

*Dr. E. C. Kirk.*—I had opportunity in 1901 to see a great deal of this diatoric work in the office of Dr. John Girdwood, of Edinburgh, who wrote a paper on it which he presented to the Columbian College in 1893. Since the advent of modern crown- and bridgework he has been developing this particular crown to that class of work. I examined a great many cases in his office in which the diatoric tooth was used, and I can testify to the utility of the work. A number had been in use from six to ten years, and were doing good service. All the advantages that Dr. Sanger has claimed for this class of operation were fully exemplified by the cases I saw.

The question has been raised as to the value of the sulphur cement. Much depends upon the accuracy with which the sulphur is used, and all the joining should be carefully done. Close adaptation requires but a small amount of sulphur to bring about union. The method is a very old one. The point which Dr. Sanger brought out, which seems to be an improvement upon the method advocated by Dr. Girdwood, is his swaging the base of the plate. Dr. Girdwood has done that by grinding, which takes more time and is not so accurate. I am very glad to see the diatoric tooth taking root, as it were, in America, because it is a very valuable tooth and adjustable to a great many conditions, and the results obtained are artistic.

*Dr. H. C. Register.*—I am pleased with Dr. Sanger's method of using the diatoric tooth. It is an advance in the right direction,—anticipating a possible breakage and restoration of a crown without disturbing the base support.

The principle Dr. Sanger has given us is about the same as that I have used in my practice. I commenced bridge-work, I suppose, nearly twenty years ago, and one of the first objects sought was to make it of such a character that in case of accident a crown could be easily replaced. I am still experimenting. The teeth I have handed to Dr. Sanger shows the *evolution* of my experimentation, ultimately discontinuing the use of a pin tooth for one pinless, using instead of pins a grooved staple formed around the approximal and gingival surface, thus forming a cup or cups by the staple and backing; these united at their approximo-occlusal portion, leaving the necks or base spaced, so that the tongue movements can sluice that portion, form the base. The articulated teeth are taken out and the metal portion finished to a perfect contour. I believe this crown is *universal in its* adaptation for every kind and position of prosthetic work,—bridge-, crown-, or plate,—which is easily replaced in case of accident without in any way moving the fixture. If a break happens after the piece has been set, the remaining portion of porcelain is taken out and a new crown slipped into the cup and cemented to place. Teeth of this character have been used with but rare breakage for eighteen years. The field is open to a more natural form and artistic arrangement of bridge- and crown-work, in which, in case of accident, the crowns can be replaced with little or no trouble. Either of these crowns meet the condition. I called the attention of the profession to this crown in its crude development many years ago. I spoke then of having a small saddle and of restricting the saddle to the tooth itself. While this is a helpful support, it also gives conciousness of the tactile faculty. I have found it clean, and it reproduces the contour line. The enunciation is better and the whole mouth restoration complete. The principal involved in these two kinds of tooth-crowns is much the same, with the favor of strength, I think, being on the side of a tooth being dove-tailed into its base.

Its use will be limited to the real artist; the "dental jeweller" wants something easier.

*Dr. J. H. Gaskill.*—Since the primary object seems to be to prevent the appearance of gold, I would like to ask Dr. Sanger why he has gold at the neck of the tooth?

*Dr. Sanger.*—I feared that in the downward pressure the pin might not sustain the weight; but I believe the lower part of that cap is not necessary. In practice the teeth are set so well up on the gum that the gold is not in evidence. I have not as yet found a bridge where it was a serious objection. (Replying to a member.) I have not been troubled with discoloration. I find that the life of the tooth is longer than if covered with a cap of any kind which would subsequently induce decay. My experience with open-faced crowns has been disastrous. Leakage occurs without loosening, and by the time the patient finds it out, the entire palatine surface of that tooth is decayed and you lose the entire crown. That is more liable than when a pin is made and when a minimum amount of gold is used. I think the Carmichael and the Marshall systems would come under the head of open-faced crowns; yet the systems are so recent I would hesitate about putting myself on record as condemning either of them before another trial. They are as yet practically new.

*Dr. Register.*—This tooth that I have shows there is no necessity of running the gold on the labial side.

*Dr. James Truman.*—I have not much to say about crowning or diatoric teeth; but, I want to add my observation of the fact that we seem to be approaching a period of more artistic dentistry than I have seen in the past. Therefore it is with peculiar gratification that I listened to Dr. Sanger speak of his application of diatoric teeth, and especially of his mode of attaching them by means of sulphur. I used it many years ago in fastening the old tube-teeth upon plates, and I have never yet found any teeth which you could possibly separate without melting the sulphur. It is better than oxyphosphate.

In my reading of the dental work advocated throughout the country I find that there is a tendency to the belief that pulps can be destroyed indiscriminately without any harm to the teeth. I think it is time that a warning should be sent out that this is an error. The statement has been made by some high in authority that the tooth is just as good after the pulp has been destroyed, that it is simply a formative organ, and therefore the sooner gotten rid of the better. If there ever was a wrong idea promulgated, it seems to me that is one. You cannot destroy the pulp without destroying a large portion of the vitality of the tooth and rendering it more liable to the influences which must subsequently act. I do not want to discuss the subject, but I think this discussion should not go out without some protest against the destruction of pulps as practised by the great majority of dentists. I do not speak of Dr. Sanger's custom. He is probably justified in his practice, but there are many who destroy pulps where it is not necessary.

*Dr. Sanger* (closing).—I seem to have occupied the floor about four-fifths of the time already, though I want to thank you for the reception you have given the paper. I want to thank Dr. Truman for disabusing your minds of the idea that might have been engendered that I believe in the indiscriminate destruction of pulps. I do not think that at any time I said that, and certainly I do not practise it. I only said that when it was indicated as a desirable course, then I would pursue the method described as pressure anæsthesia. Certainly no one recognizes more than I do the intrinsic value of the pulp. I believe most heartily that any living body is better than one that is half dead.

After some discussion on Incidents of Practice, the meeting adjourned.

Otto E. Inglis,
*Editor Academy of Stomatology.*

